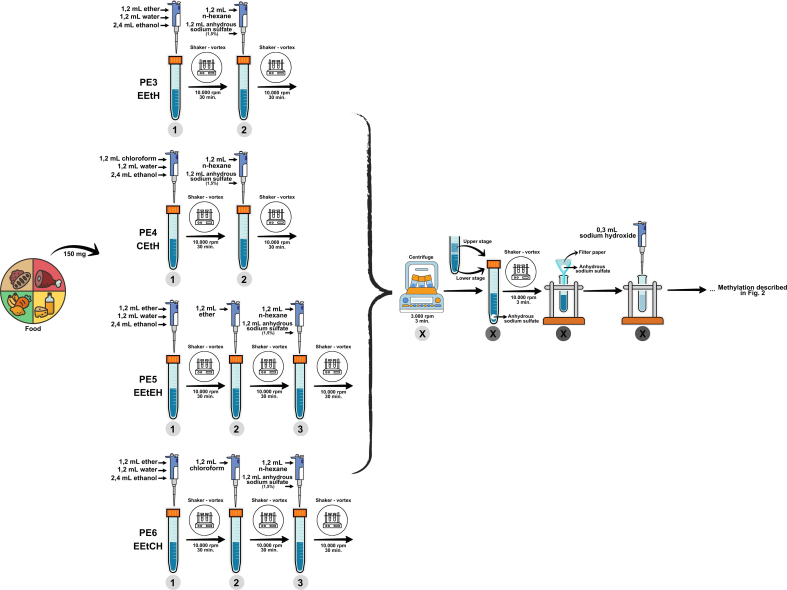# Fat extraction methodology can affect the food fatty acid profile: Improvement of a protocol more environmentally friendly

**DOI:** 10.1016/j.heliyon.2024.e40206

**Published:** 2024-11-09

**Authors:** Wemerson de Castro Oliveira, Thiago Freitas Soares, Neila Silvia Pereira dos Santos Richards, Maria Beatriz Prior Pinto Oliveira

**Affiliations:** a Department of Education, Research and Extension, Federal Institute of Education, Science and Technology Sul-rio-grandense, Lajeado, 95910-016, Brazil; b REQUIMTE/LAQV, Department of Chemical Sciences, Faculty of Pharmacy, University of Porto, Porto, 4050-313, Portugal; c Department of Food Science and Technology, Federal University of Santa Maria, Santa Maria, 97105-900, Brazil

**Keywords:** MUFA, PUFA, Food quality, Green chemistry, Extraction protocol, GC-FID

## Abstract

Fatty acids (FAs) are biochemical components of food, essential for human health due to their numerous biological functions. However, many of then if consumed in excess can trigger disfunctions/illness. Therefore, analytical methods, such as gas chromatography (GC-FID) are essential for the accurate identification and quantification of FAs, playing an important role in food safety and quality assessment. The aim of this study was to compare two FAs extraction protocols and to adapt, optimize, and validate a new one for food FAs extraction, considering the principles of green chemistry (sustainability and environmentally friendly). The research included four main steps: (A) comparison of two FAs extraction protocols for Gas Chromatography Flame Ionization Detector (GC-FID) analysis; (B) adaptation and optimization of four new extraction protocols; (C) verification of the most promising protocol; and (D) evaluation of the greenness profile. Comparatively, the PE2 protocol detected a greater diversity of FAs and a wider range of medium/long chain saturated fatty acids (SFA), but with divergences from values declared on food labels, mainly in the quantification of unsaturated fatty acids (UFA), unlike PE1, which presented lipid fraction values closer to nutritional information and greater extraction of short/medium chain FAs. The improved protocol, PE6, proved to be more efficient in extracting short, medium, and long chain FAs, as well as having SFA and UFA values closer to nutritional values in different food matrices. Furthermore, PE6 also showed improvements in relation to green chemistry aspects, presenting a higher greenness profile score, which indicates that it is more sustainable and generates less environmental impact. These results underscore the importance and relevance of continuing to develop analytical methods for food that are both effective and environmentally responsible.

## Highlights

• Comparison of two fatty acid extraction protocols for cheese analysis.
• Standardization, optimization, and verification of a fatty acid extraction protocol.
• Faster, more efficient, and less environmentally polluting fatty acid extraction protocol.

## 1 Introduction

Fatty acids (FAs) are aliphatic monocarboxylic acids that are part of the triacylglycerols present in fat and oils and can be liberate after hydrolysis. The constitution is based on hydrocarbon chains with 2–30 or more carbon atoms, a reactive carboxyl group forming ester bonds, and a methyl group at the opposite end, which can be abbreviated by the number of carbon atoms, the number of double bonds, and the position of the first double bond. The carbon chain of this chemical structure can be categorized based on the number of carbon atoms, such as short-chain FA (SCFA), with less than 6 carbon atoms, medium-chain FA (MCFA), with less than 13 carbon atoms, and long-chain FA (LCFA) that have 13 or more carbon atoms [1]. Another existing classification, which is the most commonly used, relates to the bond types: single bonds, called saturated (SFA), and double bonds, called unsaturated (UFA), including monounsaturated FAs (MUFA), and polyunsaturated (PUFA) according to the number of double bonds [2,3].

Several studies report that FAs perform various essential biological functions, including energy production, regulation of cellular metabolism, membrane structure, intracellular signaling, immunity, and inflammation (notably chronic inflammation in the human body). Additionally, FAs are antiatherogenic, enhance human cognitive functions, improve insulin secretion, increase apoptosis in cancer cells, and help prevent adverse coronary events [[3], [4], [5], [6], [7], [8], [9], [10], [11]].

The benefits of these FAs, described previously, are related to the size of the carbon chain in the molecule. SCFA and MCFA contribute to the regulation of cellular metabolism and play an important role in intracellular signaling [10]. Current studies have shown that MCFAs are beneficial for infants, as they are quickly metabolized by the human body and serve as a quick source of energy, enhancing gluconeogenesis [9,12]. Furthermore, FAs with a chain between 6 and 8 carbon atoms mediate allergic inflammation and regulate the absorption of electrolytes and water from the gastrointestinal tract, allowing MUFAs to potentially influence intestinal flora and immune function [12]. Roopashree et al. [9] found that MCFA can boost immune response and insulin secretion by activating G protein-coupled receptors. This activation may also promote apoptosis in cancer cells and help manage metabolic diseases by altering the gut microbiota. The health benefits of LCFAs, particularly omega 3 (n-3), have been extensively studied, demonstrating beneficial physiological effects [13], such as reducing the risk of developing cardiovascular diseases [14] and inflammatory processes. Additionally, omega-3, -6, and -9 FAs are extremely important in the diet due to diet, contributing to improvements in mental and cardiac health, reducing low-density lipoprotein (LDL) cholesterol, and decreasing inflammation [15,16].

A balanced intake of PUFA is essential for maintaining health. Since no single oil can provide all nutritional needs, it is recommended to use a combination of edible oils to obtain an optimized ratio of ω-6/ω-3 PUFA. The ratio between PUFA and SFA is commonly used as an index to assess the impact of a diet on cardiovascular health [17]. This index suggests that all PUFA present in the diet can reduce low-density lipoprotein cholesterol (LDL-C) and total cholesterol levels in the blood, while SFAs are associated with increased serum cholesterol [17]. Therefore, a higher PUFA/SFA ratio is associated with better health outcomes [17]. Studies have found that a ω-6/ω-3 PUFA ratio of 5:1 or lower increases PPAR-γ (peroxisome proliferator-activated receptor) expression, reduces NF-κB (nuclear factor-κB) activity, and decreases reactive oxygen species (ROS) production in the aorta, in addition to reducing total cholesterol levels. These factors collectively improve lipid metabolism, reduce inflammation, and reduce oxidative stress [18,19]. The World Health Organization (WHO) recommends a dietary ratio of 1.5:1:1 for SFAs/MUFAs/PUFAs and a ratio of 5–10:1 for ω-6/ω-3 PUFA in edible oil consumption [20].

Several parameters in fats and oils have received increasing interest from the scientific community due to their importance for food quality and safety. They also help to determine the authenticity, type, geographic origin of foods, and in guiding industrial processing [13,[21], [22], [23]]. An example of this is the assessment of the free FA content in crude oils, which is used to characterize high-quality oils, such as extra virgin olive oil, to check for damage to olives, and to investigate fish that contain beneficial fats (ω-3 or ω-6) [13].

Foods, such as fermented dairy products, have a complex and dynamic matrix, both at biochemical and microbiological levels, which makes the traceability and quality control of these products difficult, requiring sophisticated analytical techniques combined with multivariate analysis [22]. The complexity of FAs imposes methodological challenges for their characterization in the food matrix, which are related to the appropriate choice of the fat extraction protocol, the availability of Fatty Acid Methyl Ester (FAME) standards, and analytical techniques [24]. However, techniques such as gas chromatography (GC) with flame ionization (FID) or mass spectrometer (MS) detectors have been used successfully and have become increasingly attractive for the identification and quantification of FAs [1,[24], [25], [26], [27], [28]]. However, to obtain accurate and reliable results using GC, an appropriate process for extracting the lipid fraction is necessary.

Recent advances in FAs extraction techniques and solvent formulation had improved the efficiency to obtain the lipid fraction in foods. Methods such as solvent extraction (used for isolation and characterization of FAs), homogeneous liquid-liquid, and Soxhlet extraction (for characterizing FAs and authenticating quality) are widely used for this purpose [[29], [30], [31], [32], [33]]. Several methods are reported to extract and analyze FAs [32,[34], [35], [36], [37], [38], [39]]. However, the majority of them have limitations, either because: they are time-consuming and not suitable for routine investigation; they involve several and time-consuming heating and evaporation steps; they extract only a fraction of the total lipids; or they require large and inconvenient volumes of non-green solvent such as hexane, chloroform and others [34].

Therefore, this research compares two protocols for FAs extraction and adapts, optimizes, and validates another protocol for this proposal in foods taking into account the principles of green chemistry.

## 2 Materials and methods

### 2.1 Food samples and chemicals

A variety of processed foods was acquired in local stores in Porto - Portugal, and analyzed in the Department of Chemical Sciences, Faculty of Pharmacy of the University of Porto. Nine different foods-goat cheese (GC), sheep cheese (SC), cow's cheese (CC), tuna (TU), hamburger (HG), vegan sausage (VS), cookie (CK), potato chips (PC) and coffee (CF) - were finely crushed in a laboratory mill (Grindomix GM200, Retsh, Haan, Germany) for 5–10 s at 10,000 rpm and stored at −80 °C till the experimental period. The food matrices were thawed at room temperature before analysis. The vegetable oil from the tuna sample was drained for 1 h before sample preparation. The adaptation, standardization and optimization of the experimental protocols were carried out in three replications and the verification experiment on different foods in five replications.

Ultra-pure water was obtained from a Milli-Q purification system (Millipore, Bedford, MA, USA). All chemicals and reagents were of analytical grade. Absolute ethanol, sulfuric acid (H_2_SO_4_), sodium hydroxide (NaOH), n-hexane, and anhydrous sodium sulfate (Na_2_SO_4_) were obtained from Merck (Darmstadt, Germany). Boron trifluoride (BF_3_) in methanol solution and the standard of Supelco 37 FAME Mix, were acquired from Sigma-Aldrich (St. Louis, MI, USA). Potassium hydroxide (KOH) was purchased from Panreac (Barcelona, Spain). Methanol was acquired from Honeywell International, Inc. (Morris Plains, NJ, USA). Sodium chloride (NaCl) and chloroform were acquired from VWR Chemicals (Alfragide, Portugal). Petroleum ether was purchased from Carlo Erba Reagents (Val de Reuil, France). Dichloromethane were acquired from Honeywell l Riedel-de Haën TM (Seelze, Germany).

### 2.2 Fatty acids extraction protocols

In this study, two FAs extraction protocols used in foods, as described by Melo et al. [32] and Bligh & Dyer [34], identified as PE1 and PE2, respectively, were comparatively evaluated. Semiquantitative analysis was initially performed on samples of goat, sheep, and cow cheese, chosen for their high lipid content and diversity. The Melo et al. [32] protocol was replicated exactly as described, while the Bligh & Dyer [34] protocol was modified to reduce sample and reagent volumes. For this adaptation, 150 mg of sample was mixed with 1.2 mL of chloroform, 1.2 mL of deionized water, and 2.4 mL of methanol, and then vortexed (Heidolph Multi Reax, Schwabach, Germany) for 30 min at 10,000 rpm.

Following vortexing, 1.2 mL of chloroform and 1.2 mL of anhydrous sodium sulfate solution in deionized water (1.5 %) were added. The mixture was then homogenized and centrifuged at 5000 rpm for 3 min (Heraeus Megafuge 16, Thermo Scientific, Waltham, Massachusetts). The lower phase was transferred to a new tube containing anhydrous sodium sulfate, shaking it for approximately 1 min by vortexing with subsequent transfer of the supernatant to another tube containing a funnel with filter paper and anhydrous sodium sulfate.

The filtrate then received approximately 150 μL of NaOH (0.4 mol/L in methanol), with subsequent stirring, followed by a heating step at 100 °C in a thermoblock (Stuart SBH130D/3, Staffordshire, United Kingdom) for a period of 10 min to begin the methylation process. After cooling in an ice bath for 5 min, 450 μL of sulfuric acid (1 mol/L in methanol) was added, and the sample was stirred and heated again at 100 °C for 10 min. After cooling in an ice bath, 1.2 mL of n-hexane was added, followed by a stirring step, and 800 μL of the upper phase was transferred to the vial for chromatographic analysis.

### 2.3 GC-FID analysis

The analysis of the FAs profile was conducted using a gas chromatograph coupled with a flame ionization detector (GC-FID, Shimadzu GC-2010 Plus, Shimadzu, Tokyo, Japan) and a split/splitless AOC-20i auto-injector (Shimadzu, Tokyo, Japan), following the procedures outlined by Melo et al. [32]. FAME separation was performed on a CP-Sil 88 silica capillary column (50 m × 0.25 mm i.d.; 0.20 μm film thickness, Varian, Middelburg, The Netherlands), with helium as the carrier gas. The temperature program used was: 120 °C for 5 min; increase to 160 °C at 2 °C/min; hold for 15 min; and increase to 220 °C at 2 °C/min. Injector and detector were at 250 °C and 270 °C, respectively. A split ratio of 1:50 and an injection volume of 1.0 μL were used. FAME identification was carried out by comparing their retention times with those of standards (Supelco 37 Component FAME Mix, Supelco, Bellefonte, PA, USA). The data analysis was based on peak areas, with results expressed as the relative percentage of each FA.

### 2.4 Adapted protocols

Considering the principles of green analytical chemistry, the objective of this work was to adapt, optimize, and standardize a protocol by combining characteristics from both protocols mentioned in section 2.2. The new protocol aims to be faster, more efficient, and able to extract the major total lipid fraction, whether saturated or unsaturated. To this end, tests were carried out to reduce the quantities of solvents (reduction varied by about 50 %, depending on the solvent, substitutions and protocol), replace polluting solvents with less harmful ones, reducing toxicity, and modify the time and steps involved.

Tests were conducted in two stages, based on the protocol described by Bligh & Dyer [34] for goat cheese: (A) replacement of organic solvents – substituting methanol with ethanol and chloroform with petroleum ether; and (B) the addition of a re-extraction step. Consequently, the following extractions were performed: (PE3) ether + ethanol-hexane (EEtH); (PE4) chloroform + ethanol-hexane (CEtH); (PE5) ether + ethanol-ether-hexane (EEtEH); (PE6) ether + ethanol-chloroform-hexane (EEtCH) (Fig. S1).

### 2.5 Verification of the PE6 protocol

For the protocol that demonstrated the highest efficiency, both verification and verification were conducted to assess its FAs extraction capacity across nine different foods (item 2.1) with varying lipid contents and profiles. Verification compared the extracted FAs percentages (both saturated and unsaturated) against those reported in the nutritional tables on food labels.

### 2.6 Greenness profile

The greenness profile of the methods mentioned above as well as the one proposed in this article was evaluated using the AGREE methodology. The AGREE score, which can vary between 0.00 and 1.00, was obtained using the AGREE-metric technique [40]. In this technique, the amount and toxicity of the reagents the waste generated, the number of steps in the sample processing, automation and miniaturization, as well as the energy value required in all stages are some of the requirements of this assessment, which is grouped into 12 different green chemistry assessment criteria [41,42]. The software Analytical Greenness Calculator (version 0.5 beta, Gdansk University of Technology, Gdansk, Poland, 2020) was used to obtain the scores [41].

### 2.7 Statistical analysis

GraphPad Software (version 8.2, Inc., La Jolla, CA, USA) was used to perform the statistical analysis of the Bland-Altman test with 95 % significance. In addition, multivariate analysis of principal components (PCA) and mean comparison by ANOVA (95 % significance) were also performed using the Excel program (XLSTAT 2023.2.0.1411).

## 3 Results

### 3.1 Comparison of extraction protocols

**Fig. 1:**
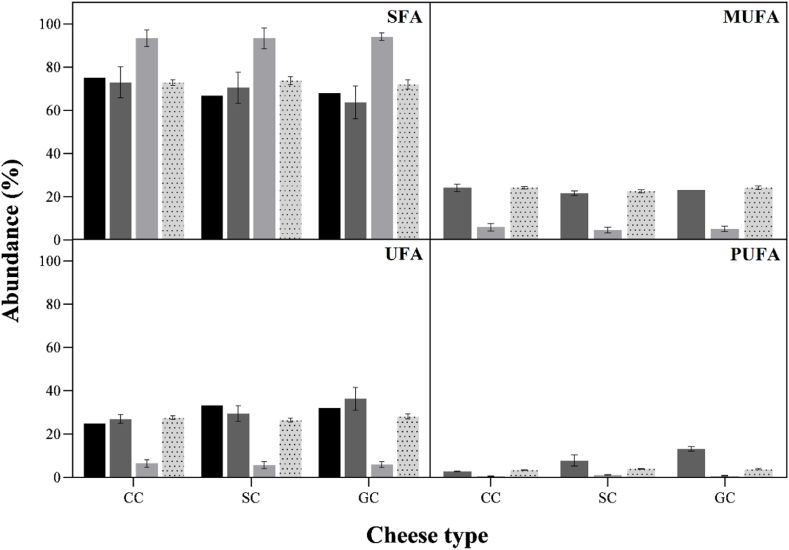
Fatty acid composition of cow (CC), sheep (SC), and goat cheese (GC) samples across three different extraction protocols. Black bars for label information, dark gray for Protocol 1(PE1), light gray for Protocol 2 (PE2), and dotted light gray for Protocol 6 (PE6).

Cheese FAs profile is of paramount importance, as a chemical biomarker for geographical origin and curing time assessing. Additionally, it is also a crucial parameter in the evaluation of the nutritional value of the food [43,44]. To obtain this profile, a step for fat extraction is needed, which can influence and generate different results, according to the used reagents and the number of steps of the process [32,34]. The FAs profile of cow, goat, and sheep cheese samples are presented in Fig. 1 and Table 1.

**Table 1:** Fatty acid profile of cow, sheep, and goat cheese samples for the two different extractive methodologies.

Fatty acids	Cow cheese (%)		Sheep cheese (%)		Goat cheese (%)	
	PE1∗	PE2∗	PE1∗	PE2∗	PE1∗	PE2∗
Butyric acid (C4:0)	3.82 ± 1.45	3.20 ± 0.12	3.04 ± 1.07	3.83 ± 0.20	1.83 ± 1.26	2.65 ± 0.09
Caproic acid (C6:0)	2.95 ± 1.10	2.43 ± 0.08	3.02 ± 0.43	3.21 ± 0.16	2.06 ± 0.79	3.17 ± 0.09
Caprylic acid (C8:0)	1.69 ± 0.49	1.71 ± 0.05	2.94 ± 0.49	3.13 ± 0.14	2.25 ± 0.37	3.87 ± 0.11
Capric acid (C10:0)	3.78 ± 0.65	4.42 ± 0.13	8.38 ± 1.74	9.52 ± 0.47	7.43 ± 0.87	13.32 ± 0.28
Undecylic acid (C11:0)	0.09 ± 0.01	0.14 ± 0.01	0.08 ± 0.01	0.12 ± 0.01	0.09 ± 0.03	0.17 ± 0.03
Lauric acid (C12:0)	4.10 ± 0.21	5.26 ± 0.14	4.02 ± 0.20	5.28 ± 0.26	3.45 ± 0.04	5.50 ± 0.07
Tridecylic acid (C13:0)	0.95 ± 0.10	0.17 ± 0.01	0.06 ± 0.03	0.10 ± 0.00	0.08 ± 0.02	0.09 ± 0.01
Myristic acid (C14:0)	11.68 ± 0.42	15.63 ± 0.40	9.34 ± 0.76	13.13 ± 0.61	8.27 ± 0.46	12.12 ± 0.21
Myristoleic acid (C14:1)	1.12 ± 0.17	0.34 ± 0.07	0.08 ± 0.04	0.06 ± 0.01	0.09 ± 0.03	0.05 ± 0.01
Pentadecylic acid (C15:0)	1.03 ± 0.03	0.97 ± 0.08	0.75 ± 0.08	1.10 ± 0.05	0.63 ± 0.04	0.89 ± 0.00
Palmitic acid (C16:0)	34.93 ± 0.87	46.64 ± 1.47	27.78 ± 1.73	38.97 ± 1.85	25.71 ± 2.21	36.01 ± 0.57
Palmitoleic acid (C16:1)	1.68 ± 0.08	0.39 ± 0.09	0.75 ± 0.08	0.28 ± 0.03	0.73 ± 0.04	0.17 ± 0.03
Margaric acid (C17:0)	0.08 ± 0.09	0.66 ± 0.01	0.40 ± 0.26	0.73 ± 0.03	0.45 ± 0.02	0.59 ± 0.02
Stearic acid (C18:0)	7.88 ± 1.87	12.02 ± 0.37	10.76 ± 0.37	14.77 ± 0.70	11.32 ± 1.45	15.52 ± 0.04
Elaidic acid (C18:1n9t)	–	0.85 ± 0.07	–	1.13 ± 0.26	0.25 ± 0.16	0.76 ± 0.13
Oleic acid (C18:1n9c)	21.39 ± 1.45	4.27 ± 1.41	20.78 ± 0.93	3.14 ± 1.09	21.85 ± 3.83	4.26 ± 1.04
Linoleic acid (C18:2n6c)	2.83 ± 0.21	0.33 ± 0.10	3.00 ± 0.19	0.69 ± 0.09	3.21 ± 0.54	0.38 ± 0.13
α-Linolenic acid (C18:3n3)	–	0.04 ± 0.00	0.20 ± 0.07	0.07 ± 0.05	0.26 ± 0.01	0.02 ± 0.00
Arachidic acid (C20:0)	–	0.12 ± 0.01	–	0.28 ± 0.04	0.17 ± 0.05	0.17 ± 0.14
Dihomo-γ-linolenic acid (C20:3n6)	–	–	–	–	0.05 ± 0.01	–
Heneicosylic acid (C21:0)	–	–	–	0.05 ± 0.00	–	–
Behenic acid (C22:0)	–	0.05 ± 0.01	–	0.07 ± 0.01	–	0.06 ± 0.00
Erucic acid (C22:1n9)	–	0.02 ± 0.00	–	0.05 ± 0.00	0.17 ± 0.06	–
Docosahexaenoic acid (C22:6n3)	–	0.31 ± 0.02	4.64 ± 2.32	0.17 ± 0.14	9.65 ± 0.48	0.32 ± 0.02
Tricosylic acid (C23:0)	–	0.05 ± 0.01	–	0.09 ± 0.01	–	0.04 ± 0.01
Lignoceric acid (C24:0)	–	0.14 ± 0.08	–	–	–	–

SFA	72.98 ± 7.09	93.46 ± 3.72	70.54 ± 7.17	93.37 ± 4.89	63.74 ± 7.58	94.05 ± 1.70
MUFA	24.19 ± 1.71	5.87 ± 1.65	21.61 ± 1.07	4.55 ± 1.39	23.10 ± 4.13	5.23 ± 1.21
PUFA	2.83 ± 0.20	0.67 ± 0.13	7.84 ± 2.57	1.08 ± 0.18	13.16 ± 1.05	0.72 ± 0.15
UFA	27.02 ± 1.91	6.54 ± 1.78	29.45 ± 3.65	5.63 ± 1.57	36.26 ± 5.18	5.95 ± 1.36

SFA (nutritional table)	75.00		66.79		68.05	
UFA (nutritional table)	25.00		33.21		31.95	

PE1- (Melo et al. (2021); PE2- (Bligh & Dyer, 1959)						

From Fig. 1 it is evident the high contents of SFA in all cheese samples. Inversely, low levels of UFA are present in the same samples with very low PUFA levels. In general, cow cheese is richer in SFAs than the other two types of cheese (goat and sheep). Both protocols PE1 and PE6 gave results more like the label composition available.

Table 1 shows some differences between the FAs profiles. The identified compounds are different, with an average of 18.3 FAs for all cheeses in PE1 and around 23.3 FAs in the PE2. In the case of PE1, taking into account all cheeses, 12 saturated FAs were identified (C4:0, C6:0, C8:0, C10:0, C11:0, C12:0, C13:0, C14:0, C15:0, C16:0, C17:0 and C18:0), 3 monounsaturated (C14:1, C16:1 and C18:1n9c) and 4 polyunsaturated FAs (C18:2n6c, C18:3n6c, C20:4n6 and C22:6n3). In the case of PE2 17 SFAs (C4:0, C6:0, C8:0, C10:0, C11:0, C12:0, C13:0, C14:0, C15:0, C16:0, C17:0, C18:0, C20:0, C21:0, C22:0, C23:0 and C24:0), 5 MUFAs (C14:1, C16:1, C18:1n9t, C18:1n9c and C22:1n9) and 3 PUFAs (C18:2n6c, C18:3n3, C22:6n3) were detected.

Despite the differences mentioned above, in both protocols, the more abundant identified FAs were hexadecanoic (palmitic, C:16:0), octadecanoic (stearic, C18:0) and tetradecanoic (myristic, C14:0) acids. These results, when taking into account those obtained by PE1, are consistent with those presented in the literature [[45], [46], [47]]. As previous referred about Fig. 1 and results obtained with PE1, cow cheese is the one with more SFAs (73.0 % determined – 75.0 % nutritional table) than goat (63.7–68.1 %) and sheep cheese (70.5–66.8 %). Considering the PE2 values all samples presented 93.5–94.1 % of SFAs, values that deviate from the labelled ones and make the samples very similar. The same conclusion can be seen for UFAs, with values ranging from 5.6 to 6.5 %. The performance of this protocol is not in accordance with scientific references and label description.

In what concerns to SCFAs (C4:0 and C6:0) goat cheese showed the lower values in PE1 (3.9 %) and cow cheese the higher ones (6.8 %). The values obtained with PE2 are different with cow cheese the poorest and the sheep cheese the richest. Butyric acid (C4:0) has higher content in cow cheese but caproic acid (C6:0) is more abundant in sheep cheese, with some differences between protocols.

In what concerns MCFAs (C8:0, C10:0, C11:0, C12:0 and C13:0) both protocols allow to extract different amounts of this type of fatty acids. The lower and similar values were determined in cow cheese (10.6–11.7 %). PE1 and PE2 exhibited worse performance in the case of the other cheeses under evaluation. The higher difference between protocols was obtained in the case of goat cheese with 13.3 and 23.0 % respectively for PE1 and PE2.

The results for LCFAs from these two protocols also showed different percentages. The FAs in this group have more than 14 carbon atoms and includes the more abundant in the studied samples, C14:0 (miristic acid), C16:0 (palmitic acid), C18:0 (stearic acid) and C18:1n9c (oleic acid). Comparatively the showed behavior differs with the type of FAs. In the case of SFA, PE2 presented higher values but with MUFA the values were lower than the obtained with PE1.

The results are consistent with those presented in the literature, also noting higher values of capric acid in these two cheeses compared to cheese from other animals [43,46,47].

One major problem observed in PE2 is that, despite the diversity of identified FAs, it conducted an incorrect semi-quantitative analysis of these compounds by providing a value for the sum of UFA much lower than the values listed in the nutritional table of these cheeses, which differs from the observations made for PE1 (Fig. 1). The MUFA and PUFA profile was similar to that described by Szterk et al. [48] for the three types of cheese, cow, sheep and goat. The authors found a small difference between the MUFAs values between the three types of cheese, lowest and highest values of 15.2 and 23.4 % for sheep's and cow's cheese, respectively. For PUFAs, the reported values varied between 1.9 and 4.3 % [48].

Comparing the two protocols, it is possible to discern distinct characteristics and advantages for each process. PE2 features fewer steps (total of 18), making the extraction process faster and more suitable for laboratory-scale analysis, while also capable of extracting a greater diversity of FAs, even at low concentrations (less than 1 %). However, the values obtained for SFA and UFA differed from those presented on the food label. The opposite was observed with PE1, where the values obtained from the lipid extraction process closely matched those listed on the label, albeit with less diversity. With the rising environmental awareness in the scientific community, consideration of analysis processes environmental impact is crucial. From this perspective, PE1 stood out a more sustainable analysis procedure compared to PE2, as it utilizes solvents and processes that are less harmful to the environment. Additional advantages and disadvantages of both protocols are detailed in Table 2.

**Table 2:** Comparison among FAs extraction protocols in three cheese samples.

Protocols	Solvents	No. of steps	Fat acids	Comments
PE1 Ferreira et al. (2023)	n-hexane; dichloromethane; ethanol.	30	**Cow:** 16–12 saturated and 4 unsaturated (3 mono and 1 poly); **Sheep:** 18 - 12 saturated and 6 unsaturated (3 mono and 3 poly); Goat: 22 - 13 saturated and 9 unsaturated (5mono and 4 poly).	Less polluting solvents; Environmentally greener extraction∗; More difficult execution; High execution time; <FAs diversity; > extraction of short/medium chain FAs; > extraction percentage (quantity); Evaporation of the solvent (nitrogen stream); Not recommended for laboratory routine; Not recommended for large-scale extraction; Values close to those on the labels.
PE2 Bligh e Dyer (1959)	chloroform; methanol; sulfuric acid; n-hexane.	18	**Cow:** 24–16 saturated and 8 unsaturated (5 mono and 3 poly); **Sheep:** 24 - 16 saturated and 8 unsaturated (5 mono and 3 poly); Goat: 22 - 15 saturated and 7 unsaturated (4 mono and 3 poly).	More polluting solvents; Less environmentally green extraction∗; Simple execution; Low execution time; > FAs diversity; > extraction of long-chain FAs; < extraction percentage (quantity) – some with a percentage below the 1 % limit; Suitable for laboratory routine; Suitable for large-scale extraction; Different values to those on the labels.
PE6 EEtCH	chloroform petroleum ether; ethanol, sulfuric acid; n-hexane.	23	**Cow:** 30 - 16 saturated and 14 unsaturated (6 mono and 8 poly); **Sheep:** 32 - 17 saturated and 15 unsaturated (6 mono and 9 poly); **Goat:** 32–17 saturated and 15 unsaturated (6 mono and 9 poly).	Polluting solvents; Environmentally greener extraction∗; Simple execution; Low execution time; > FAs diversity; > extraction of long-chain FAs; < extraction percentage (quantity) – some with a percentage below the 1 % limit; Suitable for laboratory routine; Suitable for large-scale extraction; Values close to those on the labels.

∗ Based on green chemistry parameters (AGREE).

### 3.2 Adaptation of a new improved protocol

Based on the comparison between PE1 and PE2 protocols, some modifications/adaptations were implemented to develop an improved protocol. These included replacing polluting organic solvents with less harmful alternatives, reducing the amount of sample required, decreasing execution time, and efficiently extracting a diverse range of short, medium, and long-chain FAs (Fig. S1).

The first adaptation in PE2 involved replacing chloroform solvent with petroleum ether, substituting methanol with ethanol, and adding an n-hexane extraction step (a step present in PE1) thus constituting PE3. This new protocol (PE3) extracted 20 different FAs, and the percentages of SFA and UFA were close to the values on the cheese label (Table 3). However, it extracted a low diversity of LCFA, rendering it unsuitable for the complete characterization of the FAs profile in foods. In the next attempt, PE4, chloroform was maintained, methanol was replaced by ethanol and another extraction step with n-hexane was added. This approach yielded the poorest results of all the tests, extracting only 9 FAs (Table 3).

**Table 3:** Comparison of the fatty acid profile for goat cheese in relation to the different extractive methodologies proposed.

Fatty acids	Goat cheese (%)			
	PE3_EEtH	PE4_CEtH	PE5_EEtEH	PE6_EEtCH
Butyric acid (C4:0)	0.57 ± 0.08	–	1.81 ± 0.10	1.26 ± 0.28
Caproic acid (C6:0)	1.44 ± 0.02	1.65 ± 1.00	2.75 ± 0.50	1.97 ± 0.30
Caprylic acid (C8:0)	2.42 ± 0.06	13.38 ± 9.31	3.78 ± 0.79	2.70 ± 0.26
Capric acid (C10:0)	8.44 ± 0.14	5.29 ± 2.13	11.45 ± 1.46	9.42 ± 0.46
Undecylic acid (C11:0)	0.20 ± 0.02	–	–	0.08 ± 0.01
Lauric acid (C12:0)	3.45 ± 0.29	2.42 ± 0.42	4.34 ± 0.21	4.02 ± 0.10
Tridecylic acid (C13:0)	0.05 ± 0.00	–	–	0.06 ± 0.01
Myristic acid (C14:0)	7.86 ± 0.60	5.29 ± 1.44	8.49 ± 1.03	9.26 ± 0.05
Myristoleic acid (C14:1)	0.16 ± 0.05	–	–	0.10 ± 0.02
Pentadecylic acid (C15:0)	0.70 ± 0.02	–	0.99 ± 0.38	0.69 ± 0.00
Palmitic acid (C16:0)	27.81 ± 0.67	20.91 ± 3.62	24.91 ± 3.83	28.75 ± 0.29
Palmitoleic acid (C16:1)	0.76 ± 0.06	–	0.69 ± 0.09	0.61 ± 0.04
Margaric acid (C17:0)	0.40 ± 0.08	–	0.44 ± 0.25	0.44 ± 0.04
Stearic acid (C18:0)	12.94 ± 0.21	9.45 ± 0.10	11.52 ± 2.19	13.14 ± 0.12
Elaidic acid (C18:1n9t)	0.28 ± 0.04	–	–	0.05 ± 0.01
Oleic acid (C18:1n9c)	26.26 ± 0.66	33.44 ± 9.00	21.38 ± 2.61	23.13 ± 0.74
Linoelaidic acid (C18:2n6t)	–	–	–	0.33 ± 0.04
Linoleic acid (C18:2n6c)	5.44 ± 0.94	8.17 ± 0.52	2.64 ± 0.35	3.15 ± 0.17
α-Linolenic acid (C18:3n3)	0.26 ± 0.00	–	–	0.20 ± 0.03
γ-Linolenic acid (C18:3n6c)	–	–	–	0.04 ± 0.01
Arachidic acid (C20:0)	–	–	–	0.23 ± 0.04
Gondoic acid (C20:1n9)	–	–	4.82 ± 8.17	0.10 ± 0.01
Eicosadienoic acid (C20:2)	–	–	–	0.01 ± 0.00
Eicosatrienoic acid (C20:3n3)	0.60 ± 0.27	–	–	0.02 ± 0.00
Dihomo-γ-linolenic acid (C20:3n6)	–	–	–	0.03 ± 0.00
Eicosapentaenoic acid (C20:5n3)	–	–	–	0.03 ± 0.01
Heneicosylic acid (C21:0)	–	–	–	0.02 ± 0.00
Behenic acid (C22:0)	–	–	–	0.05 ± 0.02
Erucic acid (C22:1n9)	–	–	–	0.19 ± 0.03
Docosahexaenoic acid (C22:6n3)	0.14 ± 0.07	–	–	0.03 ± 0.01
Tricosylic acid (C23:0)	–	–	–	0.02 ± 0.00
Lignoceric acid (C24:0)	–	–	–	0.01 ± 0.00
SFA	66.10 ± 2.21	58.39 ± 18.01	70.47 ± 10.33	71.98 ± 2.00
MUFA	27.46 ± 0.81	33.44 ± 8.99	26.89 ± 10.87	24.18 ± 0.85
PUFA	6.44 ± 1.28	8.17 ± 0.52	2.64 ± 0.35	3.84 ±0.30
UFA	33.90 ± 2.09	41.61 ± 9.51	29.53 ± 11.22	28.02 ± 1.15
SFA (nutritional table)	68.05			
UFA (nutritional table)	31.95			

**Subtittle:** PE3_EEtH: éter + ethanol-hexano; PE4_CEtH: clorofórmio + ethanol-hexano; PE5_EEtEH: éter + ethanol-éter-hexano; PE6_EEtCH: éter + ethanol-clorofórmio-hexano.

In the next option (PE5), chloroform was replaced by petroleum ether, methanol by ethanol, addition of a re-extraction step with petroleum ether and another extraction step with n-hexane. The protocol showed efficiency in lipid extraction, with values, SFA = 70.5 % and UFA = 29.5 %, very close to the information contained in the label, SFA = 68.0 % and UFA = 32.0 %. Moreover, it is a more sustainable protocol as it uses reagents that are less harmful to the environment. It is an ideal protocol for routine laboratory use. The final protocol, PE6, used in further analyses, entailed an extraction step with petroleum ether, a substitution of methanol with ethanol, a second extraction with chloroform, and a subsequent n-hexane extraction (Fig. S1). This protocol presented the greatest diversity of FAs, totaling 32, with the SFA and UFA values (72.0 and 28.0 %, respectively) close to the label information (Fig. 1) and not different from the previous protocol (PE5).

PE6 was designed to use a reduced amount of sample (150 mg), and the protocol was chosen for verification as it is more suitable for evaluating the authenticity and adulteration of food, as it extracts numerous FAs, and can thus be used as a tool to determine the Protected Designation of Origin (PDO) or Geographic Identification (GI) of a food. An example of this is the research carried out by Borges et al. [49], who evaluated the FAs profile of chestnuts from 17 cultivars, for two years in three PDO regions in Portugal and detected variations in lipid composition between cultivars, indicating differences in genotypes. Other authors also address this topic in their research [13,[21], [22], [23]] and point out the importance of using chromatographic techniques equipped with several detectors for the identification of chemical markers of adulteration, such as FA. This type of assessment has already been used in the analysis of adulteration of dairy products and oil [[50], [51], [52]].

In short, this protocol (PE6) is more environmentally sustainable, considering the parameters of green chemistry, and is easy and quick to apply on a laboratory analysis scale. It allows the simultaneous extraction of many samples and is the most suitable among those tested to confirm the authenticity and characterization of FAs from food matrices. Comparatively, it presents more advantages for this type of analysis. As disadvantage of this protocol is the use of chloroform, a polluting solvent.

**Fig. 2:**
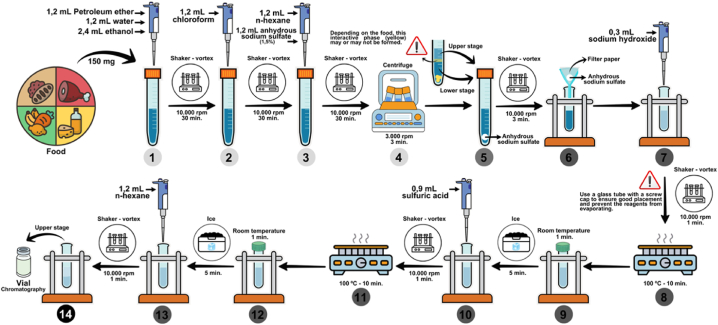
New protocol for FAs extraction from foods. (PE6) ether + ethanol-chloroform-hexane (EEtCH). In the extraction of some foods, three phases did not occur as indicated in the illustration (step 5). In foods in which three phases were observed, the decomposition of food residues was noted, which did not precipitate during centrifugation and were stored between the two solvent phases. In step 14, if residue is observed in the upper phase, a centrifugation for 5 min at 3000 rpm was performed. Note: In the extraction of some foods, three phases did not occur as indicated in the illustration (step 5). In foods in which three phases were observed, the decomposition of food residues was noted, which did not precipitate during centrifugation and were stored between the two solvent phases. In step 14, if residue is observed in the upper phase, centrifuge for 5 min at 3000 rpm.

The protocol, detailed in Fig. 2, begins by weighing 150 mg of sample, followed by the addition of 1.2 mL of petroleum ether, 1.2 mL of water and 2.4 mL of ethanol (step 1) and stirring for 30 min (10,000 rpm). Subsequently, 1.2 mL of chloroform was added and stirred again, under the same conditions, for another 30 min (step 2). After the stirring period, 1.2 mL of n-hexane and 1.2 mL of anhydrous sodium sulfate solution in deionized water (1.5 %) were added and stirred for another 30 min at 10,000 rpm (step 3), then centrifuged at 3000 rpm for 3 min (step 4). The upper and lower phases were transferred to another tube^1^ containing anhydrous sodium sulfate (approximately 200 mg) (step 5), shaken for 3 min (10,000 rpm), and filtered through a paper filter containing approximately 200 mg of anhydrous sodium sulfate (step 6). Next, 0.3 mL of sodium hydroxide (0.4 mol/L in methanol) was added to the filtrate (step 7), stirred for 1 min (10,000 rpm), heated at 100 °C for 10 min (step 8), cooled for 1 min at room temperature and 5 min in an ice bath (step 9). Afterwards 0.9 mL of sulfuric acid (1 mol/L in methanol) were added (step 10), stirred for 1 min (10,000 rpm), heated at 100 °C for 10 min (step 11), cooled for 1 min at room temperature and 5 min in an ice bath (step 12), add 1.2 mL of n-hexane (step 13), shaked for 1 min (10,000 rpm) and 0.8 mL transferred to the vial.

**Fig. 3:**
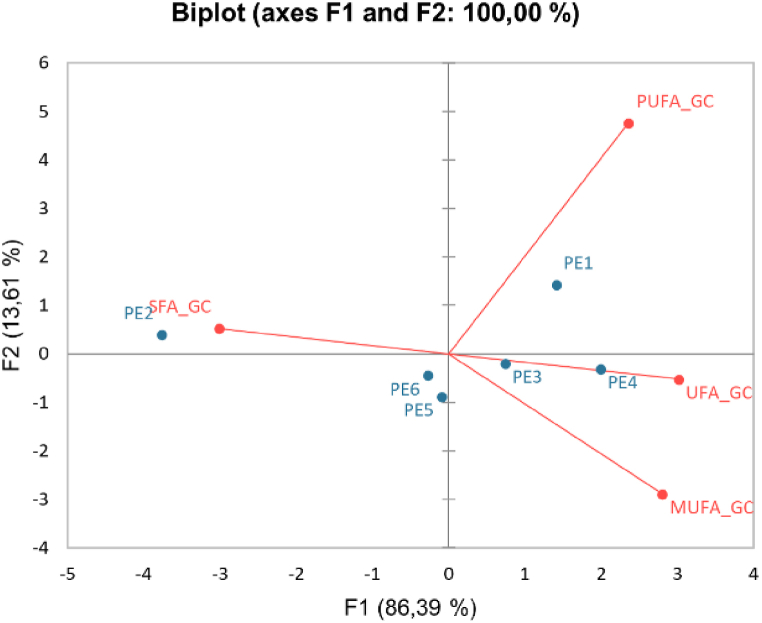
Principal component analysis (PCA) of goat cheese FAs profile. Variables: saturated (SFA), unsaturated (UFA), monounsaturated (MUFA) and polyunsaturated (PUFA) fatty acids and observations: different extraction protocols. (PE3) ether + ethanol-hexane (EEtH); (PE4) chloroform + ethanol-hexane (CEtH); (PE5) ether + ethanol-ether-hexane (EEtEH); (PE6) ether + ethanol-chloroform-hexane (EEtCH).

Fig. 3 is a PCA from goat cheese samples and shows the correlation between the different types of FAs and the six protocols employed in the extraction process. In PC1, accounting for 86.4 %, the abundance of SFA is inversely proportional to the UFA (MUFA and PUFA). The protocols were separated into two groups based on the predominant type of FA extracted; PE2 extracts more SFA while PE4 extracts more UFA. Protocols PE5 and PE6 are closer to the central axis (zero), indicating its ability to efficiently extract both saturated and unsaturated FAs.

In PC 2, PE1 is closer to PUFA, and along with PE2, it is inversely proportional to the composition of the other FAs types. Notably, the PE6 protocol aligns closer to zero in both Cartesian planes, PC 1 and 2, suggesting a higher extraction efficiency of the analyzed samples. It emerges as the most suitable protocol among those tested to determine the FAs profile in cheeses.

### 3.3 Protocol verification

**Fig. 4:**
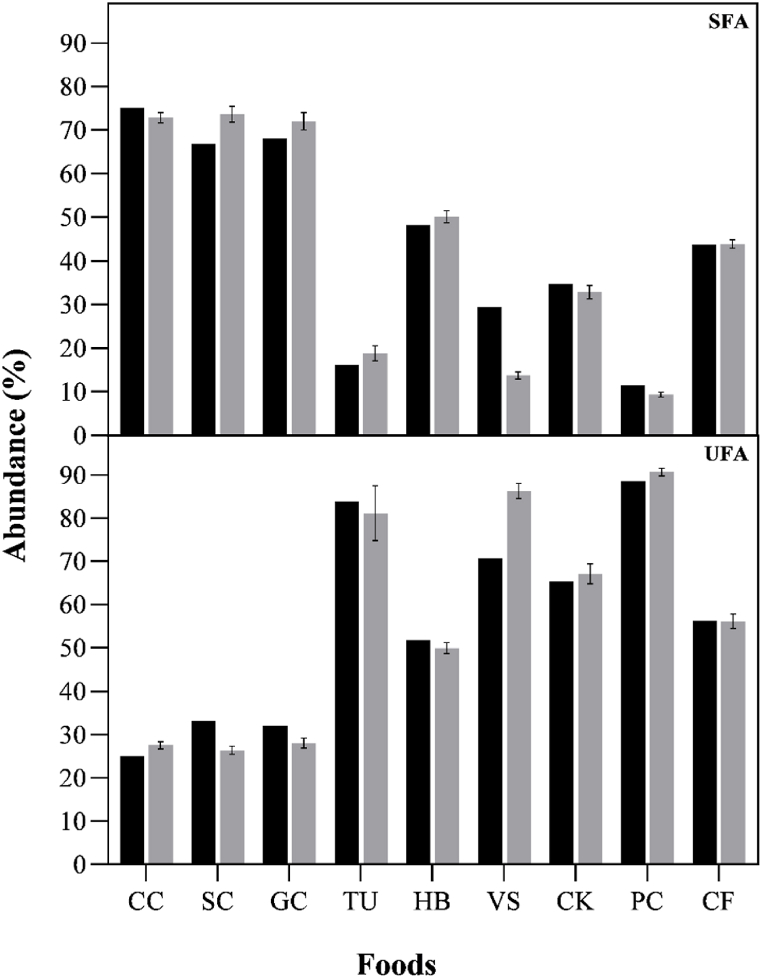
Comparison of the FAs profile of foods. (SFA) saturated fatty acids and (UFA) unsaturated fatty acids. Visualization is represented by bars: black for label information, and light gray for Protocol 6 (PE6). This analysis encompasses various foods: cow's cheese (CC), sheep cheese (SC), goat cheese (GC), tuna (TU), hamburger (HB), vegan sausage (VS), cookies (CK), potato chips (PC), and coffee (CF).

Fig. 4 is the graphical representation of the FAs composition of 9 foodstuff samples determined by EP6 and the reported values on food labels. It is observed that the SFA and UFA are very close to those listed on the food labels. From all evaluated foods, vegan sausage exhibited the most significant variation and the largest discrepancy in extraction.

Table 4 summarizes the FAs profile of only 8 foodstuffs (less 1cheese sample) using PE6 protocol. The diversity of FAs extracted varied according to the analyzed sample, being detected 19 FAs in potato chips and 32 FAs in sheep cheese. Interestingly, the proposed method successfully extracted FAs with small, medium, and large chains. As referred above identified SFA and UFA are very close to those listed on the food label. Notably, the food sample showing the smallest difference was CF (0.18 % SFA higher), while the largest difference for SFA was in the VS sample (15.60 % lower). This behavior can be attributed to the low total lipids content in vegan sausage which can interfere with the extraction process. Only 3 samples presented lower SFAs values than the labelled (vegan sausage, cow cheese and cookies), meaning that these foods had higher quality than the labelled, because SFAs are correlated with undesirable health effects if consumed in excess.

**Table 4:** Fatty acid profile of food samples based on the optimized extractive methodology (PE6).

Fatty acids	Foods (%)							
	Tuna	Hamburger	Vegan Sausage	Cow cheese	Sheep cheese	Cookie	Potato chips	Coffee
Butyric acid (C4:0)	–	–	–	1.92 ± 0.17	2.09 ± 0.05	–	–	–
Caproic acid (C6:0)	–	0.02 ± 0.01	0.02 ± 0.00	1.68 ± 0.10	2.17 ± 0.03	0.03 ± 0.01	–	0.03 ± 0.01
Caprylic acid (C8:0)	0.05 ± 0.02	0.07 ± 0.01	0.09 ± 0.01	1.28 ± 0.08	2.23 ± 0.24	0.47 ± 0.04	0.03 ± 0.01	0.10 ± 0.03
Capric acid (C10:0)	0.08 ± 0.04	0.11 ± 0.01	0.13 ± 0.01	3.30 ± 0.12	7.10 ± 0.33	0.48 ± 0.03	0.02 ± 0.01	0.10 ± 0.03
Undecylic acid (C11:0)	0.03 ± 0.01	–	0.04 ± 0.01	0.09 ± 0.01	0.07 ± 0.00	0.02 ± 0.01	0.02 ± 0.01	0.05 ± 0.01
Lauric acid (C12:0)	0.04 ± 0.02	0.11 ± 0.01	0.11 ± 0.06	3.94 ± 0.06	4.02 ± 0.06	6.63 ± 0.35	0.03 ± 0.02	0.10 ± 0.03
Tridecylic acid (C13:0)	–	0.01 ± 0.00	–	0.13 ± 0.01	0.07 ± 0.01	–	–	–
Myristic acid (C14:0)	0.27 ± 0.07	3.53 ± 0.13	0.31 ± 0.08	12.09 ± 0.11	10.41 ± 0.15	2.67 ± 0.14	0.09 ± 0.03	0.24 ± 0.09
Myristoleic acid (C14:1)	–	1.20 ± 0.04	–	1.02 ± 0.01	0.12 ± 0.01	–	–	–
Pentadecylic acid (C15:0)	0.08 ± 0.01	0.59 ± 0.01	0.02 ± 0.01	1.14 ± 0.01	0.89 ± 0.01	0.03 ± 0.01	0.02 ± 0.01	0.05 ± 0.01
Palmitic acid (C16:0)	12.34 ± 0.96	28.93 ± 0.65	8.21 ± 0.43	37.03 ± 0.37	31.87 ± 0.56	17.31 ± 0.75	4.67 ± 0.25	33.16 ± 0.30
Palmitoleic acid (C16:1)	0.43 ± 0.04	4.69 ± 0.21	0.25 ± 0.04	1.66 ± 0.01	0.79 ± 0.02	0.17 ± 0.02	0.16 ± 0.03	0.20 ± 0.03
Margaric acid (C17:0)	0.28 ± 0.04	1.01 ± 0.03	0.05 ± 0.00	0.52 ± 0.01	0.59 ± 0.01	0.06 ± 0.01	0.03 ± 0.00	0.10 ± 0.01
Stearic acid (C18:0)	4.75 ± 0.39	15.56 ± 0.48	3.75 ± 0.12	9.49 ± 0.09	12.23 ± 0.30	4.23 ± 0.07	3.06 ± 0.05	7.04 ± 0.13
Elaidic acid (C18:1n9t)	–	0.11 ± 0.01	–	0.11 ± 0.06	0.21 ± 0.09	–	–	–
Oleic acid (C18:1n9c)	21.47 ± 1.36	41.45 ± 0.60	33.72 ± 0.42	21.11 ± 0.43	21.00 ± 0.42	57.43 ± 1.70	82.08 ± 0.60	14.86 ± 0.70
Linoelaidic acid (C18:2n6t)	–	0.28 ± 0.03	–	0.36 ± 0.02	0.38 ± 0.01	–	–	–
Linoleic acid (C18:2n6c)	49.20 ± 3.53	1.26 ± 0.17	51.62 ± 1.17	2.48 ± 0.14	2.99 ± 0.16	8.95 ± 0.47	7.86 ± 0.21	39.21 ± 0.75
α-Linolenic acid (C18:3n3)	6.54 ± 0.42	0.53 ± 0.01	0.33 ± 0.03	0.34 ± 0.01	0.37 ± 0.02	0.25 ± 0.04	0.20 ± 0.03	0.97 ± 0.02
γ-Linolenic acid (C18:3n6c)	–	–	–	0.02 ± 0.01	0.06 ± 0.01	–	–	–
Arachidic acid (C20:0)	0.46 ± 0.03	0.12 ± 0.01	0.22 ± 0.02	0.13 ± 0.02	0.23 ± 0.02	0.28 ± 0.02	0.26 ± 0.02	2.24 ± 0.11
Gondoic acid (C20:1n9)	0.22 ± 0.02	0.17 ± 0.02	0.16 ± 0.03	0.05 ± 0.01	0.08 ± 0.01	0.19 ± 0.04	0.24 ± 0.01	0.65 ± 0.06
Eicosadienoic acid (C20:2)	0.05 ± 0.01	–	–	0.03 ± 0.01	0.02 ± 0.00	–	–	0.04 ± 0.01
Eicosatrienoic acid (C20:3n3)	0.05 ± 0.02	–	0.09 ± 0.03	0.03 ± 0.01	0.02 ± 0.01	0.08 ± 0.00	0.11 ± 0.01	0.05 ± 0.02
Dihomo-γ-linolenic acid (C20:3n6)	–	0.07 ± 0.01	–	0.12 ± 0.01	0.03 ± 0.00	–	–	–
Eicosapentaenoic acid (C20:5n3)	0.38 ± 0.12	0.05 ± 0.01	–	–	0.02 ± 0.01	–	–	–
Heneicosylic acid (C21:0)	–	0.03 ± 0.00	–	0.02 ± 0.01	0.05 ± 0.01	–	–	0.04 ± 0.01
Behenic acid (C22:0)	0.33 ± 0.05	0.03 ± 0.01	0.65 ± 0.03	0.05 ± 0.01	0.09 ± 0.01	0.478 ± 0.02	0.84 ± 0.05	0.38 ± 0.04
Erucic acid (C22:1n9)	0.28 ± 0.15	0.08 ± 0.01	0.06 ± 0.02	0.18 ± 0.02	0.23 ± 0.01	–	–	0.10 ± 0.05
Docosadienoic acid (C22:2)	–	–	–	–	–	0.05 ± 0.03	–	–
Docosahexaenoic acid (C22:6n3)	2.15 ± 0.27	0.01 ± 0.00	0.04 ± 0.02	0.01 ± 0.00	0.02 ± 0.01	0.02 ± 0.00	–	0.05 ± 0.01
Tricosylic acid (C23:0)	0.05 ± 0.01	–	–	–	0.04 ± 0.01	0.03 ± 0.01	0.03 ± 0.01	0.05 ± 0.02
Lignoceric acid (C24:0)	0.10 ± 0.01	0.02 ± 0.00	0.19 ± 0.03	0.03 ± 0.00	0.02 ± 0.01	0.21 ± 0.06	0.30 ± 0.02	0.19 ± 0.03

SFA	18.84± 1.74	50.10 ± 1.39	13.74 ± 0.82	72.81 ± 1.18	73.64 ± 1.82	32.86 ± 1.53	9.36 ± 0.55	43.88 ± 0.96
MUFA	22.40 ± 1.69	47.70 ± 1.02	34.18 ± 0.51	24.14 ± 0.60	22.46 ± 0.68	57.80 ± 1.76	82.47 ± 0.65	15.80 ± 0.85
PUFA	58.76 ± 4.67	2.20 ± 0.26	52.08 ± 1.24	3.39 ± 0.21	3.90 ± 0.21	9.34 ± 0.55	8.16 ± 0.25	40.32 ± 0.81
UFA	81.16 ± 6.36	49.90 ± 1.29	86.26 ± 1.75	27.53 ± 0.81	26.36 ± 0.89	67.14 ± 2.31	90.64 ± 0.90	56.12 ± 1.66

SFA (nutritional table)	16.15	48.22	29.38	75.00	66.79	34.69	11.47	43.70
UFA (nutritional table)	83.85	51.78	70.62	25.00	33.21	65.31	88.53	56.30

Dairy matrices exhibited the highest amounts of SFAs (Fig. 4); inversely, vegan sausage, potato chips, and tuna, showed the highest UFAs values (Fig. 4 and Table 4). The FAs profile from tuna differed from the values reported by Peng et al. [53] in the muscle of species *Thunnus albacares* and *Thunnus obesus* (44.9 and 37.0 % for SFA, and 21.6 and 24.8 % for UFA, respectively). This difference was attributed to the presence of vegetable oil in canned tuna. García et al. [54] showed that the muscle of white tuna (*Thunnus alalunga*) absorbs the soybean oil during the sterilization process, increasing the concentration of fat, the ratio of polyunsaturated to saturated fatty acids, and the n-6/n-3 ratio. This situation reduces the levels of oleic, linoleic and linolenic acids, as well as eicosapentaenoic (EPA) and docosahexaenoic (DHA) acids. The latter, found mainly in fish, are known for their anticancer, cardioprotective and antiarteriosclerotic effects [55]. In the present study, the tuna sample analyzed presents these fatty acids in its composition, as shown in Table 4.

This finding is in line with the general results, which show that, in all food samples, the FAs oleic acid (C18:1n9c, 14.9–82.1 %) and linoleic acid (C18:2n6c, 1.3–51.6 %) are present in greater amounts. In addition to them, we also evidenced the presence of palmitic acid (C16:0, 4.7–37.0 %) and stearic acid (C18:0, 3.1–15.6 %) in greater amounts.

**Fig. 5:**
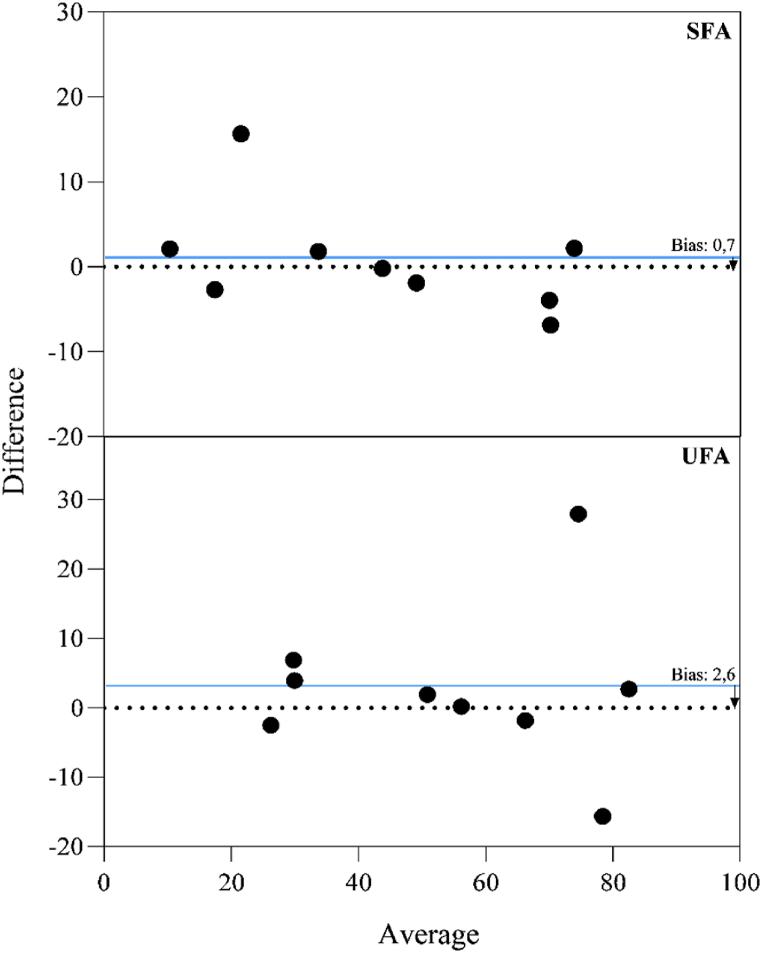
Bland-Altman plots illustrate the relationship between lipid values reported on the food label and those obtained using PE6 protocol, compared to the average of the measurements. Each closed circle represents an analyzed food item. (SFA) saturated fatty acids and (UFA) unsaturated fatty acids. The average difference is represented by the gap between the X axis, corresponding to the difference from zero, and the line parallel to the X-axis (blue line). A significance of 95 % was applied.

PE6 shows to be a satisfactory method for lipids extraction. When compared the values on food labels with those obtained by this extraction protocol, the average difference, indicated as bias above zero, is approximately 0.7 % for SFA and 2.6 % for UFA (Fig. 5). This variation is considered small, given the low sample amount used in the analysis, the challenges associated with the lipid extraction and the various food matrices with distinct lipid compositions and contents. In the extraction of SFA (Fig. 5), it was observed that only one of the 9 foods analyzed deviated significantly from the zero axis, whereas for UFA, two foods showed notable differences from the others. This demonstrates the method's efficiency and versatility in handling products from diverse categories, including dairy, meat (beef and fish), vegetable, and highly processed items (such as stuffed biscuits and potato chips).

The proposed new protocol is straightforward, requiring only a small sample amount (150 mg) and less time compared to the other protocols. It has been validated for routine lipid analysis and for studying lipid composition in food matrices with different lipid contents.

### 3.4 Greenness profile

Analytical Greenness (AGREE) is the most widely used quantitative ecological assessment methodology lately based on the verification of the 12 principles of green analytical chemistry [40,41]. This system gives weights to each of the principles, aiming to obtain more flexible and personalized specific requirements of each assessment, thereby emphasizing certain aspects in detriment of others [56]. Therefore, in order to calculate the AGREE score, each of the variables of these principles is evaluated, assigning a score to each principle. The final result is generated from the sum of the scores of all principles [56,57]. In this methodology the score varies from 0.00 to 1.00, where for a score above 0.75 it is observed that the method is excellent in relation to green chemistry, a score lower than 0.75 and equal to 0.50 demonstrates adequate greenness and, finally, a score lower than 0.50 demonstrates an inadequate method [41,57]. Concomitantly with the score value, the results are presented on a color scale to assist in the visual representation of the result. This evaluation is presented in a pictogram with a clock-shaped circle, wherein the score is available on the inside of the circle and the color varies from green to red, demonstrating that the method complies with the principles of analytical chemistry, whether green or not, respectively [40].

**Fig. 6:**
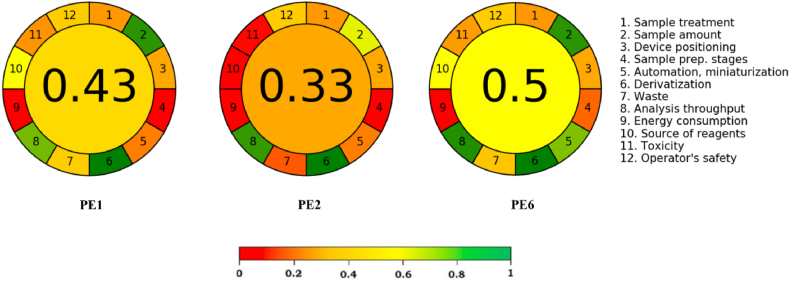
Analytical Greenness (AGREE) scale for FAs extraction by different methods.

The AGREE pictogram and the score for evaluating the FAs extraction methods reported in the literature (PE1 and PE2) together with the suggested methodology (PE6) are presented in Fig. 6. From the analysis of this pictogram, there was an improvement in the principles of green analytical chemistry, with PE6 falling within the zone of adequate greenness. Furthermore, it was the protocol with the higher capacity for FAs extraction as observed in PCA (Fig. 3). In this protocol, section 4, corresponding to the number of sample processing steps, was slightly improved, changing from a reddish color to an orange color. Another improvement generated was observed in section 5, obtaining a greenish color. As a result of these aspects, as well as some others that are not so visible, a score of 0.50 was observed for protocol 3 at the expense of 0.43 and 0.33 in relation to the other protocols, PE1 and PE2, respectively.

## 4 Conclusion

The protocols already established in the scientific community for extracting the lipid profile, PE1 and PE2, showed significant differences in relation to the quantity, number and type of fatty acids identified. The PE1 protocol was shown to be more aligned with the nutritional composition indicated on the labels, in addition to being more sustainable compared to PE2, due to the use of less harmful solvents.

Modifications were made to the protocols, replacing polluting solvents with less harmful alternatives, reducing the amount of sample required and decreasing the execution time, which resulted in a more efficient extraction of fatty acids. The improved protocol, PE6, demonstrated greater efficiency in the extraction of a variety of fatty acids, becoming a valuable tool for determining the Denomination of Origin of the product. In addition, the SFA and UFA values obtained with PE6 were shown to be closer to those presented on the labels of various food matrices.

In addition, the PE6 protocol stood out for its benefits in terms of green chemistry, achieving a high score in the sustainability index, which indicates a lower environmental impact. This protocol was effective in extracting FAs from all foods analyzed in the study, especially cheese and coffee. However, it was less efficient in extracting lipids from vegan sausages when compared to the other foods. In summary, PE6 is ideal for use in the laboratory and in analyses involving a larger number of samples. These results highlight the importance of continuing to develop effective and environmentally responsible analytical methods for food analysis.

## CRediT authorship contribution statement

**Wemerson de Castro Oliveira:** Writing – review & editing, Writing – original draft, Supervision, Formal analysis, Data curation, Conceptualization. **Thiago Freitas Soares:** Writing – review & editing, Validation, Methodology, Investigation, Data curation, Conceptualization. **Neila Silvia Pereira dos Santos Richards:** Writing – review & editing, Validation. **Maria Beatriz Prior Pinto Oliveira:** Writing – review & editing, Supervision, Project administration, Funding acquisition, Conceptualization.

## Data availability statement

Dataset available on request from the authors.

## Funding

This work received financial support from Fundação de Amparo à Pesquisa do Rio Grande do Sul – FAPERGS (23/2551-0000170-5) and FCT/MCTES (UIDP/50006/2020 DOI 10.54499/UIDP/50006/2020) through national funds. Grants funded: Conselho Nacional de Desenvolvimento Científico e Tecnológico (CNPq), Fundação de Amparo à Pesquisa do Rio Grande do Sul (FAPERGS) and FCT (DOI: 10.54499/LA/P/0008/2020; 10.54499/UIDP/50006/2020 and 10.54499/UIDB/50006/2020).

## Declaration of competing interest

The authors declare that they have no known competing financial interests or personal relationships that could have appeared to influence the work reported in this paper.

## Figures and Tables

**figs1:**